# Supplementary material for: Minimally Invasive Surgery and Surgical Volume-Specific Survival and Perioperative Outcome: Unmet Need for Evidence in Gynecologic Malignancy
Source: J Clin Med. 2021 Oct 19;10(20):4787. doi: 10.3390/jcm10204787 (PMC8537091; doi:10.3390/jcm10204787)
Supplement: Supplementary file 1 [file jcm-10-04787-s001.zip › jcm-1410746-supplementary.pdf]

Supplemental Table S1. PICOS criteria for inclusion of systematic review.

**Population**	Patients with malignant disease
**Intervention**	Laparoscopic surgery
**Comparison**	High volume center *versus* low volume center
**Outcome**	Surgical morbidity, mortality, cost, length of hospital stay, rate of laparotomy conversion, and rate of positive surgical margins
**Study design**	Retrospective or prospective cohort studies, case-control study, and randomized controlled trials

Abbreviations: PICOS, Patient/Population, Intervention, Comparator, Outcome, Study.

Supplemental Table S2. Summary of included studies evaluating the effect of surgical volume in minimally invasive surgery.

Author	Year	Study period	Area	No.	Robotic	Cancer type	Surgery type
**Gynecology (4 studies)**							
Matsuo K [1]	2020	2001-2011	USA	4,822	-	Ovarian Ca	Oophorectomy
Matsuo K [2]	2020	2007-2011	USA	2,202	Yes*	Cervical Ca	Radical hysterectomy
Wright JD [3]	2014	2006-2012	USA	10,906	Yes*	EM Ca	Hysterectomy
Wright JD [4]	2012	2000-2010	USA	4,137	-	EM Ca	Hysterectomy
**GI tract (9 studies)**							
Concors SJ [5]	2019	2010-2015	USA	8,107	Yes	Colorectal Ca	Colectomy
Salfity H [6]	2019	2010-2013	USA	2,371	No	Esophag Ca	Esophagectomy
Gietelink L [7]	2016	2011-2012	NLD	5,161	-	Colorectal Ca	Colectomy
Murata A [8]	2015	2009-2011	JPN	5,941	-	Gastric Ca	Gastrectomy
Zheng Z [9]	2014	2003-2007	USA	4,617	-	Colorectal Ca	Colectomy
Keller DS [10]	2013	2010-2012	USA	1,428	Yes	na	Colectomy
Kuwabara K [11]	2009	2007	JPN	3,765	-	Colorectal Ca	Colectomy
Yasunaga H [12]	2009	2006-2007	JPN	1,212	-	Colorectal Ca	Colectomy
Kuhry E [13]	2005	1997-2003	NLD	627	-	Colorectal Ca	Colectomy
**HPB (2 studies)**							
Nassour I [14]	2018	2010-2013	USA	1,623	Yes*	Pancreas Ca	PD
Adam MA [15]	2017	2000-2012	USA	865	Yes*	Pancreas Ca	PD
**GU (7 studies)**							
Xia L [16]	2020	2010-2014	USA	114,957	Yes	Prostate Ca	Radical prostatectomy
Peyronnet B [17]	2018	2009-2015	FRA	1,222	Yes	Renal tumor	Partial nephrectomy
Weiner AB [18]	2015	2010-2011	USA	87,415	Yes*	Prostate Ca	Radical prostatectomy
Monn MF [19]	2014	2009-2011	USA	17,583	Yes	Renal tumor	Partial nephrectomy
Hyams ES [20]	2013	2008-2011	USA	1,489	Yes	na	Radical prostatectomy
Yu HY [21]	2012	2008	USA	2,348	Yes	Prostate Ca	Radical prostatectomy
Budäus L [22]	2011	2005-2008	DEU	2,108	-	Prostate Ca	Radical prostatectomy
**Other (1 study)**							
Tchouta LN [23]	2017	2008-2013	USA	8,253	Yes	Lung tumor	Lobectomy

* mixed with conventional laparoscopic surgery. Abbreviations: na, not applicable; -, not specified; No., number; GYN, gynecology; HPB, Hepato-Pancreato-Biliary; GI, Gastrointestinal, GU, genitourinary; Ca, cancer; PD, Pancreaticoduodenectomy; DEU, Germany; NLD, Netherlands; FRA, France; JPN, Japan; USA, United States of America; EM, endometrial; and Esophag, Esophageal

Supplemental Table S3. Risk of bias assessment for the comparator study.

Authors	Confounding	Selection	Classification of intervention	Deviations from interventions	Missing data	Measurement of outcomes	Reported results	Overall bias
Matsuo K [1]	Moderate	Serious	Moderate	Moderate	Moderate	Moderate	Serious	Moderate
Matsuo K [2]	Moderate	Serious	Moderate	Moderate	Moderate	Moderate	Serious	Moderate
Wright JD [3]	Moderate	Serious	Serious	Moderate	No information	Low	Moderate	Moderate
Wright JD [4]	Moderate	Serious	Moderate	Moderate	No information	Moderate	Serious	Moderate
Concors SJ [5]	Moderate	Serious	Moderate	Moderate	No information	Moderate	Serious	Moderate
Salfity H [6]	Moderate	Serious	Moderate	Moderate	Moderate	Moderate	Moderate	Moderate
Gietelink L [7]	Moderate	Serious	Serious	Moderate	No information	Low	Moderate	Moderate
Murata A [8]	Moderate	Serious	Serious	Moderate	No information	Moderate	Serious	Serious
Zheng Z [9]	Moderate	Serious	Moderate	Moderate	Moderate	Moderate	Serious	Moderate
Keller DS [10]	Moderate	Serious	Serious	Moderate	No information	Moderate	Serious	Moderate
Kuwabara K [11]	Moderate	Serious	Serious	Moderate	Serious	Moderate	Serious	Serious
Yasunaga H [12]	Moderate	Serious	Serious	Moderate	No information	Moderate	Serious	Serious
Kuhry E [13]	Low	Moderate	Moderate	Low	Low	Moderate	Low	Low
Nassour I [14]	Moderate	Serious	Moderate	Moderate	No information	Moderate	Serious	Moderate
Adam MA [15]	Moderate	Serious	Moderate	Moderate	Moderate	Moderate	Serious	Moderate
Xia L [16]	Moderate	Serious	Moderate	Moderate	Moderate	Moderate	Moderate	Moderate
Peyronnet B [17]	Moderate	Serious	Moderate	Moderate	No information	Moderate	Serious	Moderate
Weiner AB [18]	Moderate	Serious	Moderate	Moderate	No information	Low	Moderate	Moderate
Monn MF [19]	Moderate	Serious	Moderate	Moderate	Moderate	Serious	Moderate	Moderate
Hyams ES [20]	Moderate	Serious	Serious	Moderate	No information	Low	Moderate	Moderate
Yu HY [21]	Moderate	Serious	Moderate	Moderate	Moderate	Moderate	Serious	Moderate
Budäus L [22]	Moderate	Serious	Serious	Moderate	No information	Low	Moderate	Moderate
Tchouta LN [23]	Moderate	Serious	Moderate	Moderate	No information	Moderate	Serious	Moderate

Risk of bias assessment was performed using the Risk Of Bias In Non-randomized Studies–of Interventions tool (ROBINS-I) [24–26].

● Low risk of bias (the study is comparable to a well-performed randomized trial with regard to this domain)

● Moderate risk of bias (the study is sound for a non-randomized study with regard to this domain but cannot be considered comparable to a well-performed randomized trial)

● Serious risk of bias (the study has some important problems in this domain)

● Critical risk of bias (the study is too problematic in this domain to provide any useful evidence on the effects of intervention.

● No information on how to base a judgment on the risk of bias for this domain.

Supplemental Table S4. Sub-analysis of volume-outcome relationship in minimally invasive surgeries for malignant diseases.

Surgery type	Author	Year	Infection	Re-operation	Hospital stay	Cost for stay	Specific age
Oophorectomy	Matsuo [1]	2020	--	--	--	--	--
PD	Nassour [14]	2018	--	--	--	--	--
	Adam [15]	2017	No	No	Yes	--	--
Radical prostatectomy	Xia [16]	2020	--	--	--	--	--
	Weiner [18]	2015	--	--	--	--	--
	Hyams [20]	2013	--	--	Yes	--	--
	Yu [21]	2012	No‡	--	Yes	--	--
	Budäus [22]	2011	--	--	Yes	--	--
Nephrectomy	Peyronnet [17]	2018	--	--	Yes	--	--
	Monn [19]	2014	--	--	Yes	--	--
Lobectomy	Tchouta [23]	2017	No	--	--	--	--
RH	Matsuo [2]	2020	--	--	--	--	--
Hysterectomy	Wright [3]	2014	--	--	--	--	--
	Wright [4]	2012	No†	--	--	--	--
Colectomy	Concors [5]	2019	--	--	--	--	--
	Gietelink [7]	2016	--	--	--	--	--
	Zheng [9]	2014	--	--	Yes	--	--
	Keller [10]	2013	No‡	--	--	--	--
	Kuwabara [11]	2009	No†	--	Yes	Yes	--
	Yasunaga [12]	2009	--	--	Yes	--	--
	Kuhry [13]	2005	No	--	Yes	--	--
Gastrectomy	Murata [8]	2015	--	--	--	--	--
Esophagectomy	Salfity [6]	2019	--	--	--	--	--

† Abdominal abscess. ‡ Wound infection.

REFERENCES

[1] Matsuo K, Chang EJ, Matsuzaki S, Mandelbaum RS, Matsushima K, Grubbs BH, et al. Minimally invasive surgery for early-stage ovarian cancer: Association between hospital surgical volume and short-term perioperative outcomes. Gynecol Oncol. 2020;158:59-65.

[2] Matsuo K, Matsuzaki S, Mandelbaum RS, Chang EJ, Klar M, Matsushima K, et al. Minimally invasive radical hysterectomy for early-stage cervical cancer: Volume-outcome relationship in the early experience period. Gynecol Oncol. 2020.

[3] Wright JD, Ananth CV, Tergas AI, Herzog TJ, Burke WM, Lewin SN, et al. An economic analysis of robotically assisted hysterectomy. Obstetrics and gynecology. 2014;123:1038-48.

[4] Wright JD, Hershman DL, Burke WM, Lu YS, Neugut AI, Lewin SN, et al. Influence of surgical volume on outcome for laparoscopic hysterectomy for endometrial cancer. Annals of surgical oncology. 2012;19:948-58.

[5] Concors SJ, Murken DR, Hernandez PT, Mahmoud NN, Paulson EC. The volume-outcome relationship in robotic protectectomy: does center volume matter? Results of a national cohort study. Surgical endoscopy. 2019.

[6] Salfity H, Timsina L, Su K, Ceppa D, Birdas T. Case Volume-to-Outcome Relationship in Minimally Invasive Esophagogastrectomy. Ann Thorac Surg. 2019;108:1491-7.

[7] Gietelink L, Henneman D, van Leersum NJ, de Noo M, Manusama E, Tanis PJ, et al. The Influence of Hospital Volume on Circumferential Resection Margin Involvement: Results of the Dutch Surgical Colorectal Audit. Ann Surg. 2016;263:745-50.

[8] Murata A, Muramatsu K, Ichimiya Y, Kubo T, Fujino Y, Matsuda S. Influence of hospital volume on outcomes of laparoscopic gastrectomy for gastric cancer in patients with comorbidity in Japan. Asian J Surg. 2015;38:33-9.

[9] Zheng Z, Hanna N, Onukwugha E, Bikov KA, Mullins CD. Hospital center effect for laparoscopic colectomy among elderly stage I-III colon cancer patients. Ann Surg. 2014;259:924-9.

[10] Keller DS, Hashemi L, Lu M, Delaney CP. Short-term outcomes for robotic colorectal surgery by provider volume. Journal of the American College of Surgeons. 2013;217:1063-9 e1.

[11] Kuwabara K, Matsuda S, Fushimi K, Ishikawa KB, Horiguchi H, Fujimori K. Impact of hospital case volume on the quality of laparoscopic colectomy in Japan. Journal of gastrointestinal surgery : official journal of the Society for Surgery of the Alimentary Tract. 2009;13:1619-26.

[12] Yasunaga H, Matsuyama Y, Ohe K. Effects of hospital and surgeon volumes on operating times, postoperative complications, and length of stay following laparoscopic colectomy. Surgery today. 2009;39:955-61.

[13] Kuhry E, Bonjer HJ, Haglind E, Hop WC, Veldkamp R, Cuesta MA, et al. Impact of hospital case volume on short-term outcome after laparoscopic operation for colonic cancer. Surgical endoscopy. 2005;19:687-92.

[14] Nassour I, Choti MA, Porembka MR, Yopp AC, Wang SC, Polanco PM. Robotic-assisted versus laparoscopic pancreaticoduodenectomy: oncological outcomes. Surgical endoscopy. 2018;32:2907-13.

[15] Adam MA, Thomas S, Youngwirth L, Pappas T, Roman SA, Sosa JA. Defining a Hospital Volume Threshold for Minimally Invasive Pancreaticoduodenectomy in the United States. JAMA surgery. 2017;152:336-42.

[16] Xia L, Sperling CD, Taylor BL, Talwar R, Chelluri RR, Raman JD, et al. Associations between Hospital Volume and Outcomes of Robot-Assisted Radical Prostatectomy. The Journal of urology. 2020;203:926-32.

[17] Peyronnet B, Tondut L, Bernhard JC, Vaessen C, Doumerc N, Sebe P, et al. Impact of hospital volume and surgeon volume on robot-assisted partial nephrectomy outcomes: a multicentre study. BJU Int. 2018;121:916-22.

[18] Weiner AB, Murthy P, Richards KA, Patel SG, Eggener SE. Population based analysis of incidence and predictors of open conversion during minimally invasive radical prostatectomy. The Journal of urology. 2015;193:826-31.
[19] Monn MF, Bahler CD, Flack CK, Dube HT, Sundaram CP. The impact of hospital volume on postoperative complications following robot-assisted partial nephrectomy. J Endourol. 2014;28:1231-6.
[20] Hyams ES, Mullins JK, Pierorazio PM, Partin AW, Allaf ME, Matlaga BR. Impact of robotic technique and surgical volume on the cost of radical prostatectomy. J Endourol. 2013;27:298-303.
[21] Yu HY, Hevelone ND, Lipsitz SR, Kowalczyk KJ, Nguyen PL, Hu JC. Hospital volume, utilization, costs and outcomes of robot-assisted laparoscopic radical prostatectomy. The Journal of urology. 2012;187:1632-7.
[22] Budaus L, Morgan M, Abdollah F, Zorn KC, Sun M, Johal R, et al. Impact of annual surgical volume on length of stay in patients undergoing minimally invasive prostatectomy: a population-based study. European journal of surgical oncology : the journal of the European Society of Surgical Oncology and the British Association of Surgical Oncology. 2011;37:429-34.
[23] Tchouta LN, Park HS, Boffa DJ, Blasberg JD, Detterbeck FC, Kim AW. Hospital Volume and Outcomes of Robot-Assisted Lobectomies. Chest. 2017;151:329-39.
[24] Sterne JA, Hernan MA, Reeves BC, Savovic J, Berkman ND, Viswanathan M, et al. ROBINS-I: a tool for assessing risk of bias in non-randomised studies of interventions. BMJ. 2016;355:i4919.
[25] Danna SM, Graham E, Burns RJ, Deschenes SS, Schmitz N. Association between Depressive Symptoms and Cognitive Function in Persons with Diabetes Mellitus: A Systematic Review. PLoS One. 2016;11:e0160809.
[26] ROBINS-I detailed guidance (2016). https://www.riskofbias.info/welcome/home/current-version-of-robins-i/robins-i-detailed-guidance-2016. (accessed 09/16/2021).
